# On two fractional differential inclusions

**DOI:** 10.1186/s40064-016-2564-z

**Published:** 2016-06-24

**Authors:** Dumitru Baleanu, Vahid Hedayati, Shahram Rezapour, Maysaa’ Mohamed Al Qurashi

**Affiliations:** Department of Mathematics, Cankaya University, Ogretmenler Cad. 14, 06530 Balgat, Ankara Turkey; Institute of Space Sciences, Magurele, Bucharest, Romania; Department of Mathematics, Azarbaijan Shahid Madani University, Azarshahr, Tabriz Iran; Department of Mathematics, King Saud University, Riyadh, Saudi Arabia

**Keywords:** Fixed point, Fractional hybrid differential inclusions, Dimension solution set

## Abstract

We investigate in this manuscript the existence of solution for two fractional differential inclusions. At first we discuss the existence of solution of a class of fractional hybrid differential inclusions. To illustrate our results we present an illustrative example. We study the existence and dimension of the solution set for some fractional differential inclusions.

## Background

As you know, fractional dynamical systems be used in modeling of some real processes and there are many published works about the existence of solutions for many fractional differential equations (see for example Baleanu et al. 2013a, b, c; Chai 2013 and the references therein) and inclusions (see for example, Benchohra and Hamidi 2010; Agarwal et al. 2013; Ahmad et al. 2013; Nieto et al. 2013; Ouahab 2008; Phung and Truong 2013; Bragdi et al. 2013 and the references therein). For finding more details about elementary notions and definitions of fractional differential equations and inclusions one can study well-known books (see for example Aubin and Ceuina 1984; Deimling 1992; Kilbas et al. 2006; Kisielewicz 1991; Podlubny 1999). Recently, it has been published many useful works about modeling of fractional differential equations via providing different applications in some fields (see for example Atangana 2016; Atangana and Alkahtani 2016; Atangana and Koca 2016a, b). In this article, we first review the existence solution for the fractional hybrid derivative inclusion $^cD^\alpha \left( \frac{y(s)}{g(s,y(s), I^{\alpha _1}y(s),\dots , I^{\alpha _n}y(s))}\right) \in G(s,y(s),I^{\beta _1}y(s),\dots , I^{\beta _k}y(s))$ with boundary conditions $y(0)=y_0$ and $y(1)=y_1$, where $1<\alpha \le 2$, $\alpha _1,\dots ,\alpha _n>0$, $\beta _1,\dots ,\beta _k>0$, $y_0, y_1 \in {\mathbb {R}}$, $^cD^\alpha$ denotes Caputo fractional derivative of order $\alpha$, $g: J\times {\mathbb {R}}^n\rightarrow {\mathbb {R}}-\{0\}$ is continuous and $G:J\times {\mathbb {R}}^k\rightarrow {\mathcal {P}}({\mathbb {R}})$ is a multifunction via some properties. Also, we review existence and dimension of the solution set of fractional derivative inclusion

$$\begin{aligned} ^{c}D^{\alpha } y(s)\in G\left( s,y(s),(\phi y)(s),(\psi y)(s),{}^{c}D^{\beta _1}y(s),\dots ,{}^{c}D^{\beta _k}y(s), I^{\gamma _1}y(s),\dots ,I^{\gamma _k}y(s)\right) \end{aligned}$$

with boundary condition $y(0)+\sum _{i=1}^k{}^{c}D^{\beta _i}y(1)+\sum _{i=1}^k I^{\gamma _i}y(1)=0$, where $0<\beta _i<\alpha \le 1$ and $0<\gamma _i<1$ for $i=1,\dots ,k$, $G:J{\times }{\mathbb {R}}^{2k+3}\rightarrow {\mathcal {P}}({\mathbb {R}})$ is a multifunction via some properties, $\gamma , \lambda :J\times J \rightarrow [0,\infty )$ are two mappings with the properties $\sup _{s\in J}|\int _0^s \lambda (s,t) ds|<\infty$ and $\sup _{s\in J}|\int _0^s \gamma (s,t) ds|<\infty$ and the functions $\phi$ and $\psi$ are defined by $(\phi y)(s)=\int _0^{s} \gamma (s,t)y(t)dt$ and $(\psi y)(s)=\int _0^{s} \lambda (s,t)y(t)dt$.

## Preliminaries

Suppose that $(\mathcal {X},d)$ be a metric space. Denote by ${\mathcal {P}}(\mathcal {X})$ and $2^\mathcal {X}$ the class of all subsets and the class of all nonempty subsets of $\mathcal {X}$ respectively. Here, ${\mathcal {P}}_{cl}(\mathcal {X})$, ${\mathcal {P}}_{bd}({\mathcal {X}})$, ${\mathcal {P}}_{cv}({\mathcal {X}})$ and ${\mathcal {P}}_{cp}({\mathcal {X}})$ denote the class of all closed, bounded, convex and compact subsets of $\mathcal {X}$ respectively. A mapping ${\mathcal {Q}}: {\mathcal {X}}\rightarrow 2^{\mathcal {X}}$ is called a multifunction on $\mathcal {X}$ and $x\in \mathcal {X}$ is called a fixed point of $\mathcal {Q}$ whenever $x\in \mathcal {Q}x$ (Deimling 1992). A multifunction ${\mathcal {Q}}:{\mathcal {X}}\rightarrow {{\mathcal {P}}}({\mathcal {X}})$ is called lower semi-continuous whenever the set ${\mathcal {Q}}^{-1}(A):=\{x\in {\mathcal {X}} : {\mathcal {Q}}x\cap A\ne \emptyset \}$ is open for each open subset *A* of $\mathcal {X}$ (Kisielewicz 1991). If the set $\{x\in {\mathcal {X}}: {\mathcal {Q}}x\subset A\}$ is open for each open set *A* of $\mathcal {X}$, then we say that $\mathcal {Q}$ is upper semi-continuous (Kisielewicz 1991). A multifunction ${\mathcal {Q}}: {\mathcal {X}}\rightarrow {\mathcal {P}}(\mathcal {X})$ is called compact whenever $\overline{\mathcal {Q}(S)}$ is a compact for each bounded subsets *S* of ${\mathcal {X}}$ (Aubin and Ceuina 1984). A multifunction ${\mathcal {Q}}: J\rightarrow {\mathcal {P}}_{cl}({\mathbb {R}})$ is said to be measurable whenever the function $s\mapsto d(y,{\mathcal {Q}}(s))=\inf \{|y-z|:z\in {\mathcal {Q}}(s)\}$ is measurable for all $y\in {\mathbb {R}}$ and $s\in J=[0,1]$ (Deimling 1992). The Pompeiu–Hausdorff metric $H=H_d$ on $2^{\mathcal {X}}\times 2^{\mathcal {X}}$ into $[0,\infty )$ is defined by $H({\mathcal {A}},{\mathcal {B}})=\max \{\sup _{a\in {\mathcal {A}}}d(a,{\mathcal {B}}), \sup _{b\in {\mathcal {B}}}d({\mathcal {A}},b)\}$, where $d({\mathcal {A}},b)=\inf _{a\in {\mathcal {A}}}d(a; b)$ (Berinde and Pacurar 2013). Then $({\mathcal {P}}_{bd,cl}({\mathcal {X}}),H)$ is a metric space and $({\mathcal {P}}_{cl}({\mathcal {X}}),H)$ is a generalized metric space (Berinde and Pacurar 2013). A multifunction ${\mathcal {Q}}:{\mathcal {X}}\rightarrow {\mathcal {P}}_{cl}({\mathcal {X}})$ is called a contraction whenever there exists $\gamma \in (0,1)$ such that $H_{d}({\mathcal {Q}}(x),{\mathcal {Q}}(y))\le \gamma d(x,y)$ for all $x,y\in \mathcal {X}$ (Covitz and Nadler 1970). Covitz and Nadler (1970) proved that each closed valued contractive multifunction on a complete metric space has a fixed point. We say that ${\mathcal {Q}}: J\times {\mathbb {R}}^{k}\rightarrow 2^{{\mathbb {R}}}$ is a Caratheodory multifunction whenever $s\mapsto {\mathcal {Q}}(s,x_1, x_{2},\ldots ,x_k)$ is measurable for all $x_1, x_{2},\ldots ,x_k\in {\mathbb {R}}$ and $(x_1, x_{2},\ldots ,x_k)\mapsto {\mathcal {Q}}(s,x_1, x_{2},\ldots ,x_k)$ is an upper semi-continuous map for almost all $s\in J$ (see Aubin and Ceuina 1984; Deimling 1992; Kisielewicz 1991). Also, a Caratheodory multifunction ${\mathcal {Q}}: J\times {\mathbb {R}}^{k}\rightarrow 2^{{\mathbb {R}}}$ is called $L^{1}$-Caratheodory whenever for each $\rho >0$ there exists $\phi _{\rho }\in L^{1}(J,{\mathbb {R}}^{+})$ such that

$$\begin{aligned} \parallel {\mathcal {Q}}(s,x_1, x_{2},\dots ,x_k)\parallel =\sup \{|v|:v\in {\mathcal {Q}}(s,x_1, x_{2},\dots ,x_k)\}\le \phi _{\rho }(s) \end{aligned}$$

for all $|x_1|, |x_{2}|,\dots ,|x_k|\le \rho$ and for almost all $s\in J$ (see Aubin and Ceuina 1984; Deimling 1992; Kisielewicz 1991).

### **Lemma 1**

(Deimling 1992) *If*$G:{\mathcal {X}}\rightarrow {\mathcal {P}}_{cl}({\mathcal {Y}})$*is upper semi-continuous, thenGr*(*G*) *is a closed subset of*${\mathcal {X}}\times {\mathcal {Y}}$. *IfGis completely continuous and has a closed graph, then it is upper semi-continuous.*

### **Lemma 2**

(Lasota and Opial 1965) *Suppose that*$\mathcal {X}$*is a Banach space,*$G: J\times {\mathcal {X}}\rightarrow {\mathcal {P}}_{cp,cv}(\mathcal {X})$*an*$L^{1}$-*Caratheodory multivalued and*$\Theta$*a linear continuous mapping from*$L^{1}(J,\mathcal {X})$*to*$C(J,\mathcal {X})$. *Then the mapping*$\Theta o S_{G}:C(J,{\mathcal {X}})\rightarrow {\mathcal {P}}_{cp,cv}C( J,{\mathcal {X}})$*defined by*$(\Theta o S_{G})(x)=\Theta (S_{G,x})$*is a closed graph mapping in*$C(J,{\mathcal {X}})\times C(J,{\mathcal {X}})$.

### **Theorem 3**

(Dhage 2006) *Suppose that*$\mathcal {X}$*is a Banach algebra space,*$S\in {\mathcal {P}}_{bd,cl,cv}(\mathcal {X})$*and*${\mathcal {A}}: S\rightarrow {\mathcal {P}}_{cl,cv,bd}(\mathcal {X})$*and*${\mathcal {B}}: S\rightarrow {\mathcal {P}}_{cp,cv}(\mathcal {X})$*two multifunctions satisfying the following conditions*

$1.\; \mathcal {A}$*is Lipschitz with a Lipschitz constantk*,

$2.\; {\mathcal {B}}$*is upper semi-continuous and compact,*

$3.\; {\mathcal {A}}x {\mathcal {B}}x$*is a convex subsetSfor all*$x\in S$,

$$4.\; Mk<1$$, *where*$$M=\Vert {\mathcal {B}}(S)\Vert =\sup \{\Vert {\mathcal {B}}x\Vert : x\in S\}.$$

*Then, there exists*$y\in S$*such that*$y\in {\mathcal {A}}y{\mathcal {B}}y$.

### **Lemma 4**

(Agarwal et al. 2013) *Suppose that*$G: [0,1]\rightarrow {\mathcal {P}}_{cp,cv}({\mathbb {R}})$*is a measurable map such that the Lebesgue measure*$\mu$*of the set*$\{s: \dim G(s)<1\}$*is zero. Then there are arbitrarily many linearly independent measurable selections*$x_1(.),\dots ,x_m(.)$*ofG*.

### **Theorem 5**

(Agarwal et al. 2013) *Suppose thatCis a nonempty closed convex subset of Banach space*$\mathcal {X}$. *Let*$G: C\rightarrow {\mathcal {P}}_{cp,cv}(C)$*b a*$\gamma$-*contraction. If*$\dim G(x)\ge m$*for all*$x\in C$, *then*$\dim Fix (G)\ge m$.

## Main results 

First, we review the fractional hybrid differential inclusion

1 $$\begin{aligned} {}^cD^\alpha \left( \frac{y(s)}{g(s,y(s), I^{\alpha _1}y(s),\dots , I^{\alpha _n}y(s))}\right) \in G\left( s,y(s),I^{\beta _1}y(s),\dots , I^{\beta _k}y(s)\right) \end{aligned}$$

with the boundary conditions $y(0)=y_0$ and $y(1)=y_1$, where $1<\alpha \le 2$, $\alpha _1,\dots ,\alpha _n>0$, $\beta _1,\dots ,\beta _k>0$, $y_0, y_1 \in {\mathbb {R}}$, ${}^cD^\alpha$ denotes Caputo fractional derivative of order $\alpha$, $g: J\times {\mathbb {R}}^n\rightarrow {\mathbb {R}}-\{0\}$ is continuous and $G:J\times {\mathbb {R}}^k\rightarrow {\mathcal {P}}({\mathbb {R}})$ is a multifunction via some properties.

### **Lemma 6**

*Suppose that*$x\in C(J, {\mathbb {R}})$, $\alpha \in (1,2]$*and*$\alpha _1,\dots ,\alpha _n>0$. *The unique solution of the fractional differential problem*${}^cD^\alpha \left( \frac{y(s)}{g(s,y(s), I^{\alpha _1}y(s),\dots , I^{\alpha _n}y(s))}\right) =x(s)$*with the boundary value conditions*$y(0)=y_0$*and*$y(1)=y_1$*is given by*

$$\begin{aligned} y(s)& = {} g(s,y(s), I^{\alpha _1}y(s),\dots , I^{\alpha _n}y(s))\\&\times \,\left[ I^\alpha x(s)+\frac{(1-s)y_0}{g(0,y(0),\underbrace{0,\ldots ,0}_{n})}+\frac{sy_1}{f(1,y(1),I^{\alpha _1}y(1),\ldots , I^{\alpha _n}y(1))}-sI^\alpha x(1)\right] . \end{aligned}$$

### *Proof*

The general solution of the equation${}^cD^\alpha \left( \frac{y(s)}{g(s,y(s), I^{\alpha _1}y(s),\dots , I^{\alpha _n}y(s))}\right) =x(s)$ is $y(s)=g(s,y(s), I^{\alpha _1}y(s),\ldots , I^{\alpha _n}y(s))\left[ I^\alpha x(s)+c_{0}+c_{1}s\right] ,$ where $c_{0}, c_{1}\in {\mathbb {R}}$ are arbitrary constants (see Kilbas et al. 2006; Podlubny 1999). By using the boundary conditions, we get $y(0)=g(0,y(0),\underbrace{0,\ldots ,0}_{n})c_{0}=y_0$ and $y(1)=g(1,y(1),I^{\alpha _1}y(1),\ldots , I^{\alpha _n}y(1))(I^{\alpha }x(1)+c_0+c_1)=y_1$. Hence, $c_0=\frac{y_0}{g(0,y(0),\underbrace{0,\ldots ,0}_{n})}$ and $c_1=\frac{y_1}{g(1,y(1),I^{\alpha _1}y(1),\ldots , I^{\alpha _n}y(1))}-I^\alpha x(1)-\frac{y_0}{g(0,y(0),\underbrace{0,\ldots ,0}_{n})}.$ This completes the proof. $\square$

$y\in {\mathcal {Y}}=C(J,{\mathbb {R}})$ is solution for the problem (1) whenever it satisfies the boundary conditions and there exists a function $v\in S_{G,y}$ such that

$$\begin{aligned} y(s)& = {} g(s,y(s), I^{\alpha _1}y(s),\dots , I^{\alpha _n}y(s))\\&\times \,\left[ I^\alpha v(s)+\frac{(1-s)y_0}{g(0,y(0),\underbrace{0,\ldots ,0}_{n})}+\frac{sy_1}{f(1,y(1),I^{\alpha _1}y(1),\ldots , I^{\alpha _n}y(1))}-sI^\alpha v(1)\right] . \end{aligned}$$

where $$S_{G,y}=\{v\in L^1[0,1]:v(s)\in G(s,y(s),I^{\beta _1}y(s),\dots , I^{\beta _k}y(s) )\,{\text{ for }} {\text{ almost }} {\text{ all} }\,s\in J \}$$.

### **Theorem 7**

*Let*$G: J\times {\mathbb {R}}^{k+1}\rightarrow {\mathcal {P}}_{cp,cv}({\mathbb {R}})$*be a Caratheodory multifunction*, $g: J\times {\mathbb {R}}^{n+1}\rightarrow {\mathbb {R}}-\{0\}$*is a continuous and bounded function with boundKand there exist continuous functions*$p, m:J\rightarrow (0,\infty )$*such that*$\Vert G(s, y_1, y_2,\ldots ,y_k)\Vert \le m(s)$*and*$|g(s,y_1, y_2,\ldots ,y_{n+1})-g(s,x_1, x_2,\ldots ,x_{n+1})|\le p(s)\sum _{i=1}^{n+1}|y_i-x_i|$*for all*$s\in J$. *If*

$$\begin{aligned}&\Vert p\Vert _\infty \left( 1+\frac{1}{\Gamma (\alpha _1+1)}+\frac{1}{\Gamma (\alpha _2+1)}+\cdots +\frac{1}{\Gamma (\alpha _n+1)}\right) \left( \frac{2\Vert m\Vert _\infty }{\Gamma (\alpha +1)}+\left| \frac{y_0}{g(0,y(0),\underbrace{0,\ldots ,0}_{n})}\right| \right. \\&\left. \quad +\,\left| \frac{y_1}{g(1,y(1),I^{\alpha _1}y(1),\ldots , I^{\alpha _n}y(1))}\right| \right) <1, \end{aligned}$$

*then the problem* (1) *has a solution.*

### *Proof*

Define $$S=\{y\in {\mathcal {Y}}: \Vert y\Vert \le L\}$$, where

$$\begin{aligned} L=K\left( \frac{2\Vert m\Vert _\infty }{\Gamma (\alpha +1)}+\left| \frac{y_0}{g(0,y(0),\underbrace{0,\ldots ,0}_{n})}\right| +\left| \frac{y_1}{g(1,y(1),I^{\alpha _1}y(1),\ldots , I^{\alpha _n}y(1))}\right| \right) . \end{aligned}$$

clearly *S* is a closed, bounded and convex subset of the Banach algebra space ${\mathcal {Y}}$. Now, consider the multivalued operators ${\mathcal {A}},{\mathcal {B}} : S\rightarrow {\mathcal {P}}({\mathcal {Y}})$ by

$$\begin{aligned} {\mathcal {A}}y(s)=\left\{ g(s,y(s), I^{\alpha _1}y(s),\ldots , I^{\alpha _n}y(s))\right\} \end{aligned}$$

and

$$\begin{aligned} {\mathcal {B}}y(s)& = {} \left\{u\in {\mathcal {Y}}:\,{\text{ there }} {\text{ exists }}\,v\in S_{G,y}\,{\text{ such }} {\text{ that }}\,u(s)=I^\alpha v(s)+\frac{(1-s)y_0}{g(0,y(0),\underbrace{0,\ldots ,0}_{n})}\right.\\&\left.+\,\frac{sy_1}{g(1,y(1),I^{\alpha _1}y(1),\ldots , I^{\alpha _n}y(1))}-sI^\alpha v(1) \quad {\text{ for }} {\text{ all }}\,s\in J\right\}. \end{aligned}$$

Thus, the problem (1) is tantamount to the problem $y\in {\mathcal {A}}(y){{\mathcal {B}}}(y)$. We prove that the multifunctions $\mathcal {A}$ and ${\mathcal {B}}$ well-defined the conditions of Theorem 3. Note that, the operator ${\mathcal {B}}=\theta \circ S_{G}$, where $\theta$ is the continuous linear operator on $L^{1}(J,{\mathbb {R}})$ into ${\mathcal {Y}}$ defined by

$$\begin{aligned} \theta v(s)=I^\alpha v(s)+\frac{(1-s)y_0}{g(0,y(0),\underbrace{0,\ldots ,0}_{n})}+\frac{sy_1}{g(1,y(1),I^{\alpha _1}y(1),\ldots , I^{\alpha _n}y(1))}-sI^\alpha v(1). \end{aligned}$$

Let $y\in S$ be arbitrary and $\{v_n\}$ a sequence in $S_{G,y}$. Then, $v_n(s)\in G(s,y(s),I^{\beta _1}y(s),\ldots , I^{\beta _k}y(s) )$ for almost $s\in J$. Because $G(s,y(s),I^{\beta _1}y(s),\ldots , I^{\beta _k}y(s) )$ is compact for all $s\in J$, there exists a convergent subsequence of $\{v_{n}(s)\}$ (we show it again $\{v_{n}(s)\}$) to some $v\in S_{G,y}$. Since $\theta$ is continuous, $\theta v_{n}(s)\rightarrow \theta v(s)$ pointwise on *J*. Because we will show that the convergence is uniform, we have to prove that$\{\theta v_{n}\}$ is an equi-continuous sequence. Suppose that $\tau < s \in J$. So, we have

$$\begin{aligned} |\theta v_{n}(s)- \theta v_{n}(\tau )|& \le {} \frac{\Vert m\Vert _\infty (s^{\alpha }-\tau ^{\alpha })}{\Gamma (\alpha +1)})+(s-\tau )|\frac{y_0}{g(0,y(0),\underbrace{0,\dots ,0}_{n})}\\&\quad +\frac{y_1}{g(1,y(1),I^{\alpha _1}y(1),\dots , I^{\alpha _n}y(1))}-I^\alpha v_n(1)|. \end{aligned}$$

Hence the right hand of above inequalities tends to 0 as $s\rightarrow \tau$ and so the sequence $\{\theta v_n\}$ is equi-continuous. By using the Arzela–Ascoli theorem, it has a uniformly convergent subsequence. Thus, there is a subsequence of $\{v_{n}\}$ (we show it again by $\{v_{n}\}$) such that $\theta v_{n}\rightarrow \theta v$. Hence, $\theta v \in \theta (S_{G ,y})$. Thus, ${\mathcal {B}}=\theta (S_{G ,y})$ is compact for all $y\in S$. Now, we show that ${\mathcal {B}}y$ is convex for all $y\in S$. Let $y\in S$ and $u, u^\prime \in {\mathcal {B}}y$. Choose $v,v^\prime \in S_{G,y}$ such that

$$\begin{aligned} u(s)=I^\alpha v(s)+\frac{(1-s)y_0}{g(0,y(0),\underbrace{0,\ldots ,0}_{n})}+\frac{sy_1}{g(1,y(1),I^{\alpha _1}y(1),\ldots , I^{\alpha _n}y(1))}-sI^\alpha v(1),\\ u'(s)=I^\alpha v'(s)+\frac{(1-s)y_0}{g(0,y(0),\underbrace{0,\ldots ,0}_{n})}+\frac{sy_1}{g(1,y(1),I^{\alpha _1}y(1),\ldots , I^{\alpha _n}y(1))}-sI^\alpha v'(1) \end{aligned}$$

for almost all $s\in J$. Let $0\le \lambda \le 1$. Then, we have

$$\begin{aligned} \lambda u(s)+(1-\lambda )u^\prime (s)& = {} \frac{1}{\Gamma (\alpha )}\int _0^s (s-t)^{\alpha -1}[\lambda v(t)+(1-\lambda ) v'(t)]ds+\frac{(1-s)y_0}{g(0,y(0),\underbrace{0,\ldots ,0}_{n})}\\&\quad +\frac{sy_1}{g(1,y(1),I^{\alpha _1}y(1),\ldots , I^{\alpha _n}y(1))}-\frac{s}{\Gamma (\alpha )}\int _0^1 (1-t)^{\alpha -1}[\lambda v(t)+(1-\lambda ) v'(t)]dt. \end{aligned}$$

Since *G* is convex valued, $\lambda u+(1-\lambda )u^\prime \in {\mathcal {B}}y$. Cleary, $\mathcal {A}$ is bounded, closed and convex valued. We prove that ${\mathcal {A}}y{\mathcal {B}}y$ is a convex subset of *S* for all $y\in S$. Suppose that $y\in S$ and $u,u'\in {\mathcal {A}}y{\mathcal {B}}y$. Choose $v,v'\in S_{G,y}$ such that

$$\begin{aligned} u(s)& = {} g\left( s,y(s), I^{\alpha _1}y(s),\ldots , I^{\alpha _n}y(s)\right) \left[ I^\alpha v(s)+\frac{(1-s)y_0}{g(0,y(0),\underbrace{0,\ldots ,0}_{n})}+\frac{sy_1}{g(1,y(1),I^{\alpha _1}y(1),\ldots , I^{\alpha _n}y(1))}-sI^\alpha v(1)\right] ,\\ u'(s)& = {} g\left( s,y(s), I^{\alpha _1}y(s),\ldots , I^{\alpha _n}y(s)\right) \left[ I^\alpha v'(s)+\frac{(1-s)y_0}{g(0,y(0),\underbrace{0,\ldots ,0}_{n})}+\frac{sy_1}{g(1,s(1),I^{\alpha _1}s(1),\ldots , I^{\alpha _n}y(1))}-sI^\alpha v'(1)\right] \end{aligned}$$

for almost all $s\in J$. Hence, we get

$$\begin{aligned} \lambda u(s)+(1-\lambda )u^\prime (s)& = {} g\left( s,y(s), I^{\alpha _1}y(s),\ldots , I^{\alpha _n}y(s)\right) \left[ \frac{1}{\Gamma (\alpha )}\int _0^s (s-t)^{\alpha -1}[\lambda v(t)+(1-\lambda ) v'(t)\right] dt\\&\quad +\frac{(1-s)y_0}{g(0,y(0),\underbrace{0,\ldots ,0}_{n})}+\frac{sy_1}{g(1,y(1),I^{\alpha _1}y(1),\ldots , I^{\alpha _n}y(1))}-\frac{s}{\Gamma (\alpha )}\int _0^1 (1-t)^{\alpha -1}[\lambda v(t)+(1-\lambda ) v'(t)]dt. \end{aligned}$$

Since *G* is convex valued, $\lambda u+(1-\lambda )u^\prime \in {\mathcal {A}}y{\mathcal {B}}y$. So, ${\mathcal {A}}y{\mathcal {B}}y$ is convex subset of ${\mathcal {Y}}$ for all $y\in {\mathcal {Y}}$. But, we have

$$\begin{aligned} |u(s)|& = {} \left| g\left( s,y(s), I^{\alpha _1}y(s),\ldots , I^{\alpha _n}y(s)\right) \left[ I^\alpha v(s)+\frac{(1-s)y_0}{g(0,y(0),\underbrace{0,\ldots ,0}_{n})}+\frac{sy_1}{g(1,y(1),I^{\alpha _1}y(1),\ldots , I^{\alpha _n}y(1))}\right. \right. \\&\left. \left. -sI^\alpha v(1)\right] \right| \le K\left( \frac{2\Vert m\Vert _\infty }{\Gamma (\alpha +1)}+\left| \frac{y_0}{g(0,y(0),\underbrace{0,\ldots ,0}_{n})}\right| +\left| \frac{y_1}{g(1,y(1),I^{\alpha _1}y(1),\ldots , I^{\alpha _n}y(1))}\right| \right) =L \end{aligned}$$

for all $s\in J$. So, $u\in S$ and ${\mathcal {A}}y{\mathcal {B}}y$ is a convex subset of *S* for all $y\in S$. Here, We show that operator ${\mathcal {B}}$ is compact. For showing this, it is enough to prove that ${\mathcal {B}}(S)$ is uniformly bounded and equi-continuous. Let $u\in {\mathcal {B}}(S)$. Choose $v\in S_{G,y}$ such that $u(s)=g(s,y(s), I^{\alpha _1}y(s),\ldots , I^{\alpha _n}y(s))\left[I^\alpha v(s)+\frac{(1-s)y_0}{g(0,y(0),\underbrace{0,\ldots ,0}_{n})}+\frac{sy_1}{g(1,y(1),I^{\alpha _1}y(1),\ldots , I^{\alpha _n}y(1))}-sI^\alpha v(1)\right]$ for some $y\in S$. Hence,

$$\begin{aligned} |u(s)|\le \left( \frac{2\Vert m\Vert _\infty }{\Gamma (\alpha +1)}+\left| \frac{y_0}{g(0,y(0),\underbrace{0,\ldots ,0}_{n})}\right| +\left| \frac{y_1}{g(1,y(1),I^{\alpha _1}y(1),\ldots , I^{\alpha _n}y(1))}\right| \right) \end{aligned}$$

and so $\Vert u\Vert _\infty =\max _{s\in J}|u(s)|\le \left(\frac{2\Vert m\Vert _\infty }{\Gamma (\alpha +1)}+|\frac{y_0}{g(0,y(0),\underbrace{0,\ldots ,0}_{n})}|+|\frac{y_1}{g(1,y(1),I^{\alpha _1}y(1),\ldots , I^{\alpha _n}y(1))}|\right).$ In this part, prove that ${\mathcal {B}}$ maps *S* to equi-continuous subsets of ${\mathcal {Y}}$. Suppose that $s, \tau \in J$ with $\tau <s$, $y\in S$ and $u \in {\mathcal {B}}y$. Choose $v\in S_{G,y}$ such that $u(s)=I^\alpha v(s)+\frac{(1-s)y_0}{g(0,y(0),\underbrace{0,\ldots ,0}_{n})}+\frac{sy_1}{g(1,y(1),I^{\alpha _1}y(1),\ldots , I^{\alpha _n}y(1))}-sI^\alpha v(1)$. Then, we have

$$\begin{aligned} |u(s)- u(\tau )|& \le {} \left( \frac{\Vert m\Vert _\infty (s^{\alpha }-\tau ^{\alpha })}{\Gamma (\alpha +1)}\right) +(s-\tau )\left| \frac{y_0}{g(0,y(0),\underbrace{0,\ldots ,0}_{n})}\right. \\&\left. \quad +\,\frac{y_1}{g(1,y(1),I^{\alpha _1}y(1),\ldots , I^{\alpha _n}y(1))}-I^\alpha v(1)\right| . \end{aligned}$$

So the right side of inequality towards to 0 as $s\rightarrow \tau$. Hence by using the Arzela–Ascoli theorem, ${\mathcal {B}}$ is compact. Here, we show that ${\mathcal {B}}$ has a closed graph. Suppose that $y_n\in S$ and $u_n\in {\mathcal {B}} y_n$ for all *n* such that $y_n\rightarrow y'$ and $u_n\rightarrow u'$. We show that $u'\in {\mathcal {B}}y'$. For each natural number *n*, choose $v_n\in S_{G,y_n}$ such that

$$\begin{aligned} u_n(s)=I^\alpha v_n(s)+\frac{(1-s)y_0}{g(0,y(0),\underbrace{0,\ldots ,0}_{n})}+\frac{sy_1}{g(1,y(1),I^{\alpha _1}y(1),\ldots , I^{\alpha _n}y(1))}-sI^\alpha v_n(1) \end{aligned}$$

for all $s\in J$. Again, consider the continuous linear operator $\theta :L^{1}(J,{\mathbb {R}})\rightarrow {\mathcal {Y}}$ such that

$$\begin{aligned} \theta (v)(s)=u(s)=I^\alpha v(s)+\frac{(1-s)y_0}{g(0,y(0),\underbrace{0,\ldots ,0}_{n})}+\frac{sy_1}{g(1,y(1),I^{\alpha _1}y(1),\ldots , I^{\alpha _n}y(1))}-sI^\alpha v(1). \end{aligned}$$

By using Lemma 2, $\theta o S_{G}$ is a closed graph operator. Since $y_n\rightarrow y'$ and $u_n\in \theta (S_{G,y_n})$ for all *n*, there is $v'\in S_{G,y'}$ such that

$$\begin{aligned} u'(s)=I^\alpha v'(s)+\frac{(1-s)y_0}{g(0,y(0),\underbrace{0,\ldots ,0}_{n})}+\frac{sy_1}{g(1,y(1),I^{\alpha _1}y(1),\ldots , I^{\alpha _n}y(1))}-sI^\alpha v'(1). \end{aligned}$$

Hence, $u'\in {\mathcal {B}}y'$. This implies that, ${\mathcal {B}}$ has a closed graph and thus the operator ${\mathcal {B}}$ is upper semi-continuous. Now, we show that $\mathcal {A}$ is a contractive multifunction. Note that,

$$\begin{aligned} H({\mathcal {A}}y,{\mathcal {A}}x)& = {} \Vert {\mathcal {A}}y-{\mathcal {A}}x\Vert =\max _{s\in J}\left| g(s,y(s), I^{\alpha _1}y(s),\ldots , I^{\alpha _n}y(s))- g(s,x(s), I^{\alpha _1}x(s),\ldots , I^{\alpha _n}x(s))\right| \\&\le \max _{s\in J}(|p(s)| |y(s)-x(s)|)\left( 1+\frac{1}{\Gamma (\alpha _1+1)}+\frac{1}{\Gamma (\alpha _2+1)}+\cdots +\frac{1}{\Gamma (\alpha _n+1)}\right) \\& = {} \Vert p\Vert _\infty \left(1+\frac{1}{\Gamma (\alpha _1+1)}+\frac{1}{\Gamma (\alpha _2+1)}+\cdots +\frac{1}{\Gamma (\alpha _n+1)}\right)\Vert y-x\Vert _\infty \end{aligned}$$

for all $x,y\in {\mathcal {Y}}$. So, $\mathcal {A}$ and ${{\mathcal {B}}}$ satisfy the conditions of Theorem 3 and thus the operator inclusions $y\in {\mathcal {A}}y {\mathcal {A}}y$ has a solution in *S*. Therefore, the problem (1) has a solution. $\square$

To illustrate our main results, we present the following example:

### *Example 1*

Here, we investigation the problem

$$\begin{aligned} {}^cD^{\frac{1}{2}}\left( \frac{y(s)}{\frac{(s+1)^2}{60}\sin y(s)+\frac{|I^{\sqrt{2}}y(s)|}{1+|I^{\sqrt{2}}y(s)|}+3}\right) \in \left[ -1, s^2 \sin y(s)+\cos (I^{\frac{1}{4}}y(s))+1\right] \,\,\,(*) \end{aligned}$$

with the boundary conditions $y(0)=\frac{\pi }{2}$ and $y(1)=0$. Put $\alpha =\frac{1}{2}$, $\alpha _1=\sqrt{2}$, $\beta _1=\frac{1}{4}$, $n=k=1$, $y_0=\frac{\pi }{2}$, $y_1=0$, $g(s,y,x)=\frac{(s+1)^2}{60}\sin y+\frac{|x|}{1+|x|}+3$, $G(s,y,x)=[-1, s^2 \sin y+\cos x +1]$, $m(s)=s^2+2$ and $p(s)=\frac{(s+1)^2}{60}$ for $s\in [0,1]$. Note that, $\Vert G(s,y,x)\Vert \le s^2+2$,

$$\begin{aligned} \left| g(s,y,x)-g(s,y',x')\right| \le \frac{(s+1)^2}{60}\left( \left| y-y'|+|x-x'\right| \right) \end{aligned}$$

and $\Vert p\Vert _\infty \left(1+\frac{1}{\Gamma (\alpha _1+1)}\right)\left(\frac{2\Vert m\Vert _\infty }{\Gamma (\alpha +1)}+|\frac{y_0}{g(0,y(0),0)}|+|\frac{y_1}{g(1,y(1),I^{\alpha _1}y(1))}|\right)=0.8784698182<1$. By using the Theorem 7, the problem $(*)$ has a solution.

Now, we review existence and dimension of the solution set of the fractional differential inclusion problem

2 $$\begin{aligned} ^{c}D^{\alpha } y(s)\in G\left( s,y(s),(\phi y)(s),(\psi y)(s),{}^{c}D^{\beta _1}y(s),\dots ,{}^{c}D^{\beta _k}y(s), I^{\gamma _1}y(s),\dots ,I^{\gamma _k}y(s)\right) \end{aligned}$$

with boundary condition $y(0)+\sum _{i=1}^k{}^{c}D^{\beta _i}y(1)+\sum _{i=1}^k I^{\gamma _i}y(1)=0$, where $0<\beta _i<\alpha \le 1$ and $0<\gamma _i<1$ for $i=1,\dots ,k$, $G:J{\times }{\mathbb {R}}^{2k+3}\rightarrow {\mathcal {P}}({\mathbb {R}})$ is a multifunction via some properties, $\gamma , \lambda :J\times J \rightarrow [0,\infty )$ are two mappings with the properties $\sup _{s\in J}|\int _0^s \lambda (s,t) ds|<\infty$ and $\sup _{s\in J}|\int _0^s \gamma (s,t) ds|<\infty$ and the functions $\phi$ and $\psi$ are defined by $(\phi y)(s)=\int _0^{s} \gamma (s,t)y(t)dt$ and $(\psi y)(s)=\int _0^{s} \lambda (s,t)y(t)dt$.

### **Lemma 8**

*Suppose that*$v\in C(J, {\mathbb {R}})$, $\alpha \in (0,1]$*and*$\beta _i,\gamma _i\in (0,1)$*with*$\alpha -\beta _i>0$*for*$1\le i\le k$. *Then solution of the problem*$^{c}D^\alpha y(s)=v(s)$*with the boundary condition*$y(0)+\sum _{i=1}^k{}^{c}D^{\beta _i}y(1)+\sum _{i=1}^k I^{\gamma _i}y(1)=0$ is

$$\begin{aligned} y(s)& = {} \frac{1}{\Gamma (\alpha )}\int _0^s(s-t)^{\alpha -1}v(t)dt+\frac{-1}{1+\sum _{i=1}^k\frac{1}{\Gamma (\gamma _i+1)}}\sum _{i=1}^k\left( \frac{1}{\Gamma (\alpha -\beta _i)}\int _0^1(1-t)^{\alpha -\beta _i-1}v(t)dt\right. \\&\left. +\,\frac{1}{\Gamma (\alpha +\gamma _i)}\int _0^1(1-t)^{\alpha +\gamma _i-1}v(t)dt\right) . \end{aligned}$$

### *Proof*

The general solution of the problem $^{c}D^\alpha y(s)=v(s)$ is formed by

$$\begin{aligned} y(s)=I^\alpha v(s)+c_0=\frac{1}{\Gamma (\alpha )}\int _0^s(s-t)^{\alpha -1}v(t)dt+c_0, \end{aligned}$$

where $c_0$ is arbitrary constant and $t\in J$ (Podlubny 1999). Thus, we obtain

$$\begin{aligned} ^cD^{\beta _i} y(s)=I^{\alpha -\beta _i} v(s)=\frac{1}{\Gamma (\alpha -\beta _i)}\int _0s(s-t)^{\alpha -\beta _i-1}v(t)dt \end{aligned}$$

and

$$\begin{aligned} I^{\gamma _i} y(s)=I^{\alpha +\gamma _i} v(s)+\frac{c_0s^{\gamma _i}}{\Gamma (\gamma _i+1)}=\frac{1}{\Gamma (\alpha +\gamma _i)}\int _0^s(s-t)^{\alpha +\gamma _i-1}v(t)dt+\frac{c_0s^{\gamma _i}}{\Gamma (\gamma _i+1)} \end{aligned}$$

for all $1\le i\le k$. By using the boundary condition, we get

$$\begin{aligned} c_0& = {} \frac{-1}{1+\sum _{i=1}^k\frac{1}{\Gamma (\gamma _i+1)}}\sum _{i=1}^k\left( \frac{1}{\Gamma (\alpha -\beta _i)}\int _0^1(1-t)^{\alpha -\beta _i-1}v(t)dt\right. \\&\left. \quad +\frac{1}{\Gamma (\alpha +\gamma _i)}\int _0^1(1-t)^{\alpha +\gamma _i-1}v(t)dt\right) . \end{aligned}$$

Hence,

$$\begin{aligned} y(s)& = {} \frac{1}{\Gamma (\alpha )}\int _0^s(s-t)^{\alpha -1}v(t)dt+\frac{-1}{1+\sum _{i=1}^k\frac{1}{\Gamma (\gamma _i+1)}}\sum _{i=1}^k\left( \frac{1}{\Gamma (\alpha -\beta _i)}\int _0^1(1-t)^{\alpha -\beta _i-1}v(t)dt\right. \\&\left. \quad +\,\frac{1}{\Gamma (\alpha +\gamma _i)}\int _0^1(1-t)^{\alpha +\gamma _i-1}v(t)dt\right) . \end{aligned}$$

This completes the proof. $\square$

An element $y\in C(J,{\mathbb {R}})$ is a solution for the problem (2) whenever it satisfies the boundary condition and there is a function $v\in L^1(J)$ such that

$$\begin{aligned} v(s)\in G(s,y(s),(\phi y)(s),(\psi y)(s),{}^{c}D^{\beta _1}y(s),\dots ,{}^{c}D^{\beta _k}y(s), I^{\gamma _1}y(s),\dots ,I^{\gamma _k}y(s)) \end{aligned}$$

for almost all $s\in J$ and

$$\begin{aligned} y(s)& = {} \frac{1}{\Gamma (\alpha )}\int _0^s(s-t)^{\alpha -1}v(t)dt+\frac{-1}{1+\sum _{i=1}^k\frac{1}{\Gamma (\gamma _i+1)}}\sum _{i=1}^k\left( \frac{1}{\Gamma (\alpha -\beta _i)}\int _0^1(1-t)^{\alpha -\beta _i-1}v(t)dt\right. \\& \quad +\,\left. \frac{1}{\Gamma (\alpha +\gamma _i)}\int _0^1(1-t)^{\alpha +\gamma _i-1}v(t)dt\right) . \end{aligned}$$

Put ${\mathcal {Y}}=\{y: y, {}^cD^{\beta _i}y\in C(J,{\mathbb {R}})\, for\,each\,i\in \{1,\dots ,k\}\}$ with the norm

$$\begin{aligned} \Vert y\Vert =\sup _{s\in J}|y(s)|+\sum _{i=1}^k\sup _{s\in J}|^cD^{\beta _i} y(s)|. \end{aligned}$$

$({\mathcal {Y}},\Vert .\Vert )$ is a Banach space (Su 2009). Define selection set of *G* at $y\in {\mathcal {Y}}$ by

$$\begin{aligned} S_{G,y}:=\left\{v\in L^{1}(J,{\mathbb {R}}):\,v(s)\in G(s,y(s),(\phi y)(s),(\psi y)(s),{}^{c}D^{\beta _1}y(s),\dots ,{}^{c}D^{\beta _k}y(s),\right.\\ \left. I^{\gamma _1}y(s),\dots ,I^{\gamma _k}y(s))\,{\text{ for }} {\text{ almost all }}\, s\in J\right\}. \end{aligned}$$

### **Theorem 9**

*Suppose that*$m\in L^1(J,{\mathbb {R}}^{+})$, $l=(\Lambda _1+\sum _{i=1}^k\Lambda ^i_2) <1$, *where*

$$\begin{aligned} \Lambda _1=\Vert m\Vert _1\left( 1+\lambda _0+\gamma _0+\sum _{i=1}^k\frac{1}{\Gamma (\gamma _i+1)}\right) \left( \frac{1}{\Gamma (\alpha )}+\frac{1}{1+\sum _{i=1}^k\frac{1}{\Gamma (\gamma _i+1)}}\sum _{i=1}^k \frac{1}{\Gamma (\alpha -\beta _i)}+\frac{1}{\Gamma (\alpha +\gamma _i)}\right) , \end{aligned}$$

$\Lambda ^i_2=\Vert m\Vert _1\left( 1+\lambda _0+\gamma _0+\sum _{i=1}^k\frac{1}{\Gamma (\gamma _i+1)}\right) \frac{1}{\Gamma (\alpha -\beta _i)}$*for*$i=1,\dots ,k$, $F: J\times {\mathbb {R}}^{2k+3}\rightarrow {\mathcal {P}}_{cv,cp}({\mathbb {R}})$*is a multifunction such that the map*$s\vdash G(s,y_1,y_2,\ldots ,_{2k+3})$*is measurable,*

$$\begin{aligned} \Vert G(s,y_1,y_2,\ldots ,_{2k+3})\Vert =\sup \{|v| : v\in G(s,y_1,y_2,\ldots ,_{2k+3})\}\le m(s) \end{aligned}$$

*and*$H(G(s,y_1,y_2,\ldots ,y_{2k+3})),G(s,x_1,x_2,\ldots ,x_{2k+3}))\le m(s)\sum _{i=1}^{2k+3}(|y_i-x_i|)$*for almost all*$s\in J$*and*$\in x_1,x_2,\ldots ,x_{2k+3}, y_1,y_2,\ldots ,y_{2k+3}\in {\mathbb {R}}$. *Then the inclusion problem* (2) *has a solution.*

### *Proof*

Note that, the multivalued map

$$\begin{aligned} s\vdash G\left( s,y(s),(\phi y)(s),(\psi y)(s),{}^{c}D^{\beta _1}y(s),\dots ,{}^{c}D^{\beta _k}y(s), I^{\gamma _1}y(s),\dots ,I^{\gamma _k}y(s)\right) \end{aligned}$$

is measurable and closed valued for all $y\in {\mathcal {Y}}$. Hence, it has a measurable selection and so the set $S_{G,y}$ is nonempty. Now, consider the operator $\Upsilon :{\mathcal {Y}}\rightarrow 2^{\mathcal {Y}}$ defined by

$$\begin{aligned} \Upsilon (y)=\left\{ \xi \in X: \,{\text{ there is }} \,v\in S_{G,y} \,:\, \xi (s)=\nu (s) \, {\text{ for all }}\, s\in J\right\} , \end{aligned}$$

where

$$\begin{aligned} \nu (s)& = {} \frac{1}{\Gamma (\alpha )}\int _0^s(s-t)^{\alpha -1}v(t)dt+\frac{-1}{1+\sum _{i=1}^k\frac{1}{\Gamma (\gamma _i+1)}}\sum _{i=1}^k\left( \frac{1}{\Gamma (\alpha -\beta _i)}\int _0^1(1-t)^{\alpha -\beta _i-1}v(t)dt\right. \\&\left. \quad +\,\frac{1}{\Gamma (\alpha +\gamma _i)}\int _0^1(1-t)^{\alpha +\gamma _i-1}v(t)dt\right) \end{aligned}$$

for all $s\in J$. Here, we prove that $\Upsilon (y)$ is a closed subset of ${\mathcal {Y}}$ for each $y\in {\mathcal {Y}}$. Let $y\in {\mathcal {Y}}$ and $\{u_n\}_{n\ge 1}$ be a sequence in $\Upsilon (y)$ with $u_n\rightarrow u$. For each *n*, choose $v_{n}\in S_{G,y}$ such that

$$\begin{aligned} u_n(s)& = {} \frac{1}{\Gamma (\alpha )}\int _0^s(s-t)^{\alpha -1}v_n(t)dt+\frac{-1}{1+\sum _{i=1}^k\frac{1}{\Gamma (\gamma _i+1)}}\sum _{i=1}^k\left( \frac{1}{\Gamma (\alpha -\beta _i)}\int _0^1(1-t)^{\alpha -\beta _i-1}v_n(t)dt\right. \\&\quad +\left. \frac{1}{\Gamma (\alpha +\gamma _i)}\int _0^1(1-t)^{\alpha +\gamma _i-1}v_n(t)dt\right) \end{aligned}$$

for almost all $s\in J$. because *G* has compact values, $\{v_n\}_{n\ge 1}$ has a subsequence which converges to a $v\in L^{1}(J,{\mathbb {R}})$. We write it again by $\{v_n\}_{n\ge 1}$. Clearly $v\in S_{G,y}$ and

$$\begin{aligned} u_{n}(s)\rightarrow u(s)& = {} \frac{1}{\Gamma (\alpha )}\int _0^s(s-t)^{\alpha -1}v(t)dt+\frac{-1}{1+\sum _{i=1}^k\frac{1}{\Gamma (\gamma _i+1)}}\sum _{i=1}^k\left( \frac{1}{\Gamma (\alpha -\beta _i)}\int _0^1(1-t)^{\alpha -\beta _i-1}v(t)dt\right. \\&\quad +\left. \frac{1}{\Gamma (\alpha +\gamma _i)}\int _0^1(1-t)^{\alpha +\gamma _i-1}v(t)dt\right) \end{aligned}$$

for all $s\in J$. This implies that $u\in \Upsilon (x)$. Thus, the multifunction $\Upsilon$ has closed values. Now, we show that $\Upsilon$ is a contractive multifunction with constant $l=(\Lambda _1+\sum _{i=1}^k\Lambda ^i_2)<1$. Suppose that $x,y\in {\mathcal {Y}}$ and $h_1\in \Upsilon (x)$. Consider $v_1\in S_{G,x}$ such that

$$\begin{aligned} h_1(s)& = {} \frac{1}{\Gamma (\alpha )}\int _0^s(s-t)^{\alpha -1}v_1(t)dt+\frac{-1}{1+\sum _{i=1}^k\frac{1}{\Gamma (\gamma _i+1)}}\sum _{i=1}^k\left( \frac{1}{\Gamma (\alpha -\beta _i)}\int _0^1(1-t)^{\alpha -\beta _i-1}v_1(t)dt\right. \\&\left. \quad +\frac{1}{\Gamma (\alpha +\gamma _i)}\int _0^1(1-t)^{\alpha +\gamma _i-1}v_1(t)dt\right) \end{aligned}$$

for almost all $s\in J$. Since

$$\begin{aligned}&H\left( G\left( s,y(s),(\phi y)(s),(\psi y)(s),{}^{c}D^{\beta _1}y(s),\dots ,{}^{c}D^{\beta _k}y(s), I^{\gamma _1}y(s),\dots ,I^{\gamma _k}y(s)\right) ,\right. \\&\left. \quad G\left( s,x(s),(\phi x)(s),(\psi x)(s),{}^{c}D^{\beta _1}x(s),\dots ,{}^{c}D^{\beta _k}x(s), I^{\gamma _1}x(s),\dots ,I^{\gamma _k}x(s)\right) \right) \\&\quad \le m(t)\left( |y(s)-x(s)|+|(\phi y)(s)-(\phi x)(s)|+|(\psi y)(s)-(\psi x)(s)|\right. \\&\qquad \left. +\sum _{i=1}^k\left| {}^{c}D^{\beta _i}y(s)-{}^{c}D^{\beta _i}x(s)\right| +\sum _{i=1}^k\left| I^{\gamma _i}y(s)-I^{\gamma _i}x(s)\right| \right) \end{aligned}$$

for almost all $s\in J$, there is

$$\begin{aligned} \nu \in \left( G\left( s,y(s),(\phi y)(s),(\psi y)(s),{}^{c}D^{\beta _1}y(s),\dots ,{}^{c}D^{\beta _k}y(s), I^{\gamma _1}y(s),\dots ,I^{\gamma _k}y(s)\right) \right) \end{aligned}$$

such that

$$\begin{aligned} |v_1(s)- \nu |& \le {} m(s)\left( |y(s)-x(s)|+|(\phi y)(s)-(\phi x)(s)|+|(\psi y)(s)-(\psi x)(s)|\right. \\&\left. \quad +\sum _{i=1}^k\left| {}^{c}D^{\beta _i}y(s)-{}^{c}D^{\beta _i}x(s)\right| +\sum _{i=1}^k\left| I^{\gamma _i}y(s)-I^{\gamma _i}x(s)\right| \right) \end{aligned}$$

for almost all $s\in J$. Define the multifunction $\Delta : J\rightarrow 2^{{\mathbb {R}}}$ by

$$\begin{aligned} \Delta (s)& = {} \left\{ \nu \in {\mathbb {R}} : |v_1(s)- \nu |\le m(s)\left( |y(s)-x(s)|+|(\phi y)(s)-(\phi x)(s)|+|(\psi y)(s)-(\psi x)(s)|\right. \right. \\&\left. \left. +\sum _{i=1}^k\left| {}^{c}D^{\beta _i}y(s)-{}^{c}D^{\beta _i}x(s)\right| +\sum _{i=1}^k\left| I^{\gamma _i}y(s)-I^{\gamma _i}x(s)\right| \right) \quad {\text{ for almost all }}\, s\in J\right\} . \end{aligned}$$

The multifunction

$$\begin{aligned} \Delta (.)\bigcap \left( G\left( .,y(.),(\phi y)(.),(\psi y)(.),{}^{c}D^{\beta _1}y(.),{}^{c}D^{\beta _2}y(.),\dots ,{}^{c}D^{\beta _k}y(.), I^{\gamma _1}y(.), I^{\gamma _2}y(.),\ldots ,I^{\gamma _k}y(.)\right) \right) \end{aligned}$$

is measurable. Thus, we can choose $v_2\in S_{G,y}$ such that

$$\begin{aligned} |v_1(s)- v_2(s)|& \le {} m(s)\left( |y(s)-x(s)|+|(\phi y)(s)-(\phi x)(s)|+|(\psi y)(s)-(\psi x)(s)|\right. \\&\left. \quad +\sum _{i=1}^k\left| {}^{c}D^{\beta _i}y(s)-{}^{c}D^{\beta _i}x(s)\right| +\sum _{i=1}^k\left| I^{\gamma _i}y(s)-I^{\gamma _i}x(s)\right| \right) \end{aligned}$$

for almost all $s\in J$. Now, define $h_2 \in \Upsilon (y)$ by

$$\begin{aligned} h_2(s)& = {} \frac{1}{\Gamma (\alpha )}\int _0^s(s-t)^{\alpha -1}v_2(t)dt+\frac{-1}{1+\sum _{i=1}^k\frac{1}{\Gamma (\gamma _i+1)}}\sum _{i=1}^k\left( \frac{1}{\Gamma (\alpha -\beta _i)}\int _0^1(1-t)^{\alpha -\beta _i-1}v_2(t)dt\right. \\&\left. \quad +\frac{1}{\Gamma (\alpha +\gamma _i)}\int _0^1(1-t)^{\alpha +\gamma _i-1}v_2(t)dt\right) . \end{aligned}$$

So

$$\begin{aligned} | h_1(s)-h_2(s)|& \le {} \frac{1}{\Gamma (\alpha )}\int _0^s(s-t)^{\alpha -1}|v_1(t)-v_2(t)|dt\\&\quad +\frac{1}{1+\sum _{i=1}^k\frac{1}{\Gamma (\gamma _i+1)}}\sum _{i=1}^k\left( \frac{1}{\Gamma (\alpha -\beta _i)}\int _0^1(1-t)^{\alpha -\beta _i-1}|v_1(t)-v_2(t)|dt\right. \\&\left. \quad +\,\frac{1}{\Gamma (\alpha +\gamma _i)}\int _0^1(1-t)^{\alpha +\gamma _i-1}|v_1(t)-v_2(t)|dt\right) \le \Lambda _1\Vert y-x\Vert \end{aligned}$$

and $|{}^{c}D^{\beta _i}h_1(s)-{}^{c}D^{\beta _i}h_2(s)|\le \frac{1}{\Gamma (\alpha -\beta _i)}\int _0^s(s-t)^{\alpha -\beta _i-1}|v_1(t)-v_2(t)|\le \Lambda ^i_2\Vert x-y\Vert$ and so we get $\Vert h_1-h_2\Vert \le (\Lambda _1+\sum _{i=1}^k\Lambda ^i_2)\Vert x-y\Vert =l\Vert x-y\Vert$. This implies that the multifunction *N* is a contraction via closed values. By using the well-known theorem of Covitz and Nadler, *N* has a fixed point which is a solution for the inclusion problem (2). $\square$

### **Lemma 10**

*Suppose that*$m\in L^1(J,{\mathbb {R}}^{+})$, $G: J\times {\mathbb {R}}^{2k+3}\rightarrow {\mathcal {P}}_{cv,cp}({\mathbb {R}})$*is a multifunction such that the map*$s\vdash G(s,y_1,y_2,\ldots ,y_{2k+3})$*is measurable,*

$$\begin{aligned} \Vert G(s,y_1,y_2,\ldots ,y_{2k+3})\Vert =\sup \{|v| : v\in G(s,y_1,y_2,\ldots ,y_{2k+3})\}\le m(s) \end{aligned}$$

*for almost all*$s\in J$*and*$\in y_1,y_2,\ldots ,y_{2k+3}\in {\mathbb {R}}$*and*$\Upsilon :{\mathcal {Y}}\rightarrow {\mathcal {P}}({\mathcal {Y}})$*is defined by*

$$\begin{aligned} \Upsilon (y)=\left\{ \xi \in {\mathcal {Y}}: \,{\text{ there is }} \,v\in S_{G,y} \,:\, \xi (s)=\nu (s) \,{\text{ for all }}\, s\in J\right\} , \end{aligned}$$

*where*

$$\begin{aligned} \nu (s)& = {} \frac{1}{\Gamma (\alpha )}\int _0^s(s-t)^{\alpha -1}v(t)dt+\frac{-1}{1+\sum _{i=1}^k\frac{1}{\Gamma (\gamma _i+1)}}\sum _{i=1}^k\left( \frac{1}{\Gamma (\alpha -\beta _i)}\int _0^1(1-t)^{\alpha -\beta _i-1}v(t)dt\right. \\&\quad +\left. \frac{1}{\Gamma (\alpha +\gamma _i)}\int _0^1(1-t)^{\alpha +\gamma _i-1}v(t)dt\right) . \end{aligned}$$

*Then*, $\Upsilon (y)\in {\mathcal {P}}_{cp.cv}({\mathcal {Y}})$*for all*$y\in {\mathcal {Y}}$.

### *Proof*

Note that the operator $\Upsilon =\theta \circ S_{G}$, where $\theta$ is the continuous linear operator on $L^{1}(J,{\mathbb {R}})$ into ${\mathcal {Y}}$ which is defined by

$$\begin{aligned} \theta v(s)& = {} \frac{1}{\Gamma (\alpha )}\int _0^s(s-t)^{\alpha -1}v(t)dt+\frac{-1}{1+\sum _{i=1}^k\frac{1}{\Gamma (\gamma _i+1)}}\sum _{i=1}^k\left( \frac{1}{\Gamma (\alpha -\beta _i)}\int _0^1(1-t)^{\alpha -\beta _i-1}v(t)dt\right. \\&\quad +\left. \frac{1}{\Gamma (\alpha +\gamma _i)}\int _0^1(1-t)^{\alpha +\gamma _i-1}v(t)dt\right) . \end{aligned}$$

Suppose that $y\in {\mathcal {Y}}$ and $\{v_n\}$ is a sequence in $S_{G,y}$. so,

$$\begin{aligned} v_n(s)\in G\left( s,y(s),(\phi y)(s),(\psi y)(s),{}^{c}D^{\beta _1}y(s),\dots ,{}^{c}D^{\beta _k}y(s), I^{\gamma _1}y(s),\dots ,I^{\gamma _k}y(s)\right) \end{aligned}$$

for almost $s\in J$. Since

$$\begin{aligned} G\left( s,y(s),(\phi y)(s),(\psi y)(s),{}^{c}D^{\beta _1}y(s),\dots ,{}^{c}D^{\beta _k}y(s), I^{\gamma _1}y(s),\dots ,I^{\gamma _k}y(s)\right) \end{aligned}$$

is compact for all $s\in J$, there exists a convergent subsequence of $\{v_{n}(s)\}$ (we show it again by $\{v_{n}(s)\}$) which converges to some $v\in S_{G,y}$. Since $\theta$ is continuous, $\theta v_{n}(s)\rightarrow \theta v(s)$ pointwise on *J*. Because we show that the convergence is uniform, we must prove that $\{\theta v_{n}\}$ is an equi-continuous sequence. Let $\tau < s \in J$. Then

$$\begin{aligned} |\theta v_{n}(s)- \theta v_{n}(\tau )|= \left| \frac{1}{\Gamma (\alpha )}\int _0^s (s-t)^{\alpha -1}v_n(t)dt-\frac{1}{\Gamma (\alpha )}\int _0^\tau (\tau -t)^{\alpha -1}v_n(t)dt\right| \\ \le \left| \frac{1}{\Gamma (\alpha )}\int _0^\tau ((s-t)^{\alpha -1}-(\tau -t)^{\alpha -1})v_n()dt|+|\frac{1}{\Gamma (\alpha )}\int _\tau ^s (s-t)^{\alpha -1}v_n(t)dt\right| . \end{aligned}$$

Note that, the right side of the inequality towards to zero when $\tau \rightarrow s$. So, the sequence $\{\theta v_n\}$ is equi-continuous and so by using the Arzela–Ascoli theorem there is a uniformly convergent subsequence. Thus, there exists a subsequence of $\{v_{n}\}$ (we show it again by $\{v_{n}\}$) such that $\theta v_{n}\rightarrow \theta v$. This implies that $\theta v \in \theta (S_{G ,y})$. Hence, $\Upsilon y=\theta (S_{G ,y})$ is compact for all $y\in {\mathcal {Y}}$. Now, we prove that $\Upsilon y$ is convex for each $y\in {\mathcal {Y}}$. Let $h, h^\prime \in \Upsilon y$. Choose $v,v^\prime \in S_{G,y}$ such that

$$\begin{aligned} h(s)& = {} \frac{1}{\Gamma (\alpha )}\int _0^s(s-t)^{\alpha -1}v(t)dt+\frac{-1}{1+\sum _{i=1}^k\frac{1}{\Gamma (\gamma _i+1)}}\sum _{i=1}^k\left( \frac{1}{\Gamma (\alpha -\beta _i)}\int _0^1(1-t)^{\alpha -\beta _i-1}v(t)dt\right. \\&\left. \quad +\,\frac{1}{\Gamma (\alpha +\gamma _i)}\int _0^1(1-t)^{\alpha +\gamma _i-1}v(t)dt\right) \end{aligned}$$

and

$$\begin{aligned} h'(s)& = {} \frac{1}{\Gamma (\alpha )}\int _0^s(s-t)^{\alpha -1}v'(t)dt+\frac{-1}{1+\sum _{i=1}^k\frac{1}{\Gamma (\gamma _i+1)}}\sum _{i=1}^k\left( \frac{1}{\Gamma (\alpha -\beta _i)}\int _0^1(1-t)^{\alpha -\beta _i-1}v'(t)dt\right. \\&\left. \quad +\frac{1}{\Gamma (\alpha +\gamma _i)}\int _0^1(1-t)^{\alpha +\gamma _i-1}v'(t)dt\right) \end{aligned}$$

for almost all $s\in J$. Let $0\le \lambda \le 1$. Then, we have

$$\begin{aligned} \lambda h(s)+(1-\lambda )h^\prime (s)& = {} \frac{1}{\Gamma (\alpha )}\int _0^s(s-t)^{\alpha -1}(\lambda v(t)+(1-\lambda )v'(t))ds\\& \quad +\frac{-1}{1+\sum _{i=1}^k\frac{1}{\Gamma (\gamma _i+1)}}\sum _{i=1}^k\left( \frac{1}{\Gamma (\alpha -\beta _i)}\int _0^1(1-t)^{\alpha -\beta _i-1}(\lambda v(t)+(1-\lambda )v'(t))dt\right. \\&\left. \quad +\,\frac{1}{\Gamma (\alpha +\gamma _i)}\int _0^1(1-t)^{\alpha +\gamma _i-1}(\lambda v(t)+(1-\lambda )v'(t))dt\right) . \end{aligned}$$

Since $S_{G,y}$ is convex, $\lambda h+(1-\lambda )h^\prime \in \Upsilon y$. This completes the proof. $\square$

One can check that the fixed point set of $\Upsilon$ is equal to the set of all solutions of the problem (2).

### **Theorem 11**

*Suppose that*$m\in L^1(J,{\mathbb {R}}^{+})$, $G: J\times {\mathbb {R}}^{2k+3}\rightarrow {\mathcal {P}}_{cv,cp}({\mathbb {R}})$*is a multifunction such that the map*$s\vdash G(s,y_1,y_2,\ldots ,y_{2k+3})$*is measurable,*

$$\begin{aligned} H\left( G(s,y_1,y_2,\ldots ,y_{2k+3}),G(s,x_1,x_2,\ldots ,x_{2k+3})\right) \le m(s)\sum _{i=1}^{2k+3}|y_i-x_i| \end{aligned}$$

*and*$\Vert G(s,y_1,y_2,\ldots ,y_{2k+3})\Vert =\sup \{|v| : v\in G(s,y_1,y_2,\ldots ,y_{2k+3})\}\le m(s)$*for almost all*$s\in J$*and*$x_1,x_2,\ldots ,x_{2k+3}, y_1, y_2, y_{2k+3} \in {\mathbb {R}}$. *If Lebesgue measure of the set*

$$\begin{aligned} \left\{ s: \dim G(s,y_1,y_2,\ldots ,y_{2k+3})<1 \,{\text{ for some }} \, y_1,y_2,\ldots ,y_{2k+3}\in {\mathbb {R}}\right\} \end{aligned}$$

*is zero and *$l<1$, *then the set of all solutions of the problem* (2) *is infinite dimensional, wherelis defined in Theorem*9.

### *Proof*

Define the operator $\Upsilon$ by

$$\begin{aligned} \Upsilon (y)=\left\{ \xi \in {\mathcal {Y}}{:} \,{\text{ there exists }} \,v\in S_{G,y} \,{\text{ such that }}\, \xi (s)=\nu (s) \,{\text{ for all }}\, s\in J\right\} , \end{aligned}$$

where

$$\begin{aligned} \nu (s)& = {} \frac{1}{\Gamma (\alpha )}\int _0^s(s-t)^{\alpha -1}v(t)dt+\frac{-1}{1+\sum _{i=1}^k\frac{1}{\Gamma (\gamma _i+1)}}\sum _{i=1}^k\left( \frac{1}{\Gamma (\alpha -\beta _i)}\int _0^1(1-t)^{\alpha -\beta _i-1}v(t)dt\right. \\&\left. \quad +\frac{1}{\Gamma (\alpha +\gamma _i)}\int _0^1(1-t)^{\alpha +\gamma _i-1}v(t)dt\right) . \end{aligned}$$

By using Lemma 10, $\Upsilon x\in {\mathcal {P}}_{cp,cv}({\mathcal {Y}})$ for each $y\in {\mathcal {Y}}$. Like to the Theorem 9, $\Upsilon$ is contraction. we show that $\dim \Upsilon y>m$ for each $y\in {\mathcal {Y}}$ and $m\ge 1$. Let $y\in {\mathcal {Y}}$ and

$$\begin{aligned} F(s)=G(s,y(s),(\phi y)(s),(\psi y)(s),{}^{c}D^{\beta _1}y(s),{}^{c}D^{\beta _2}y(s),\dots ,{}^{c}D^{\beta _k}y(s), I^{\gamma _1}y(s), I^{\gamma _2}y(s),\ldots ,I^{\gamma _k}y(s)) \end{aligned}$$

for all $s\in J$. By using Lemma 4, there exist linearly independent measurable selections $v_1,\dots ,v_m$ for *F*. Put

$$\begin{aligned} h_i(s)& = {} \frac{1}{\Gamma (\alpha )}\int _0^s(s-t)^{\alpha -1}v_i(t)dt+\frac{-1}{1+\sum _{i=1}^k\frac{1}{\Gamma (\gamma _i+1)}}\sum _{i=1}^k\left( \frac{1}{\Gamma (\alpha -\beta _i)}\int _0^1(1-t)^{\alpha -\beta _i-1}v_i(t)dt\right. \\&\left. +\frac{1}{\Gamma (\alpha +\gamma _i)}\int _0^1(1-t)^{\alpha +\gamma _i-1}v(t)_idt\right) \end{aligned}$$

for $i=1,\dots ,m$. Assume that $\sum _{i=1}^m a_i h_i(s)=0$ for almost $s\in J$. By using the Caputo derivatives, we get $\sum _{i=1}^m a_i v_i(s)=0$ for almost $s\in J$. Hence, $a_1=\dots ,a_n=0$. This implies that $h_1,\dots ,h_m$ are linearly independent. Thus, $\dim \Upsilon y\ge m$. Now by using Theorem 5, the set of fixed points of $\Upsilon$ is infinite dimensional. $\square$

## Conclusions

The existence of solution for fractional differential inclusions is an important task which can be used successfully in solving real world problems from many fields of science and engineering. Thus, in our paper we analyze firstly the existence of solution of a given class of fractional hybrid differential inclusions. An example was give in order to show the reported results Secondly we concentrate our attention on proving the existence and dimension of the solution set for some fractional differential inclusions. These results are useful for the numerical studies involving the investigated equations.
